# Notes and Formulæ

**Published:** 1889-09

**Authors:** 


### PRACTICAL NOTES AND FORMULÆ.

**Earache.**—A liniment is recommended by Paresi (*Pacific Record*) for this affection, composed of—

| | |
|---|---|
| Camphorated chloral | 5 parts. |
| Glycerine | 30 parts. |
| Oil of sweet almonds | 10 parts. |

It is applied twice daily on soft cotton, being introduced as far as possible into the ear, and may also be rubbed behind the ear. The pain is almost instantly relieved, and the inflammation in many cases is subdued. The liniment must be kept in carefully closed bottles.

One of the greatest living authorities upon buccal bacteriology, Dr. Miller, finds that by using the following mixture he can completely sterilize the mouth cavities in carious teeth, etc.: Thymol, 4 grains; benzoic acid, 45 grains; tincture eucalyptus, 3½ drachms; water, 25 fluid ounces. The mouth is to be well rinsed with the mixture, especially just before going to bed, since most of the damage by fermentative and putrefactive processes in the mouth, is done at night during sleep.—*Medical World.*

**Treatment of Burns.**—For superficial burns of the face, Dr. Christopher Heath, Holme Professor of Clinical Surgery in University College, London, recommends (*Lancet*, May 25, 1889) a mixture of:

| | |
|---|---|
| Collodion | part 1. |
| Castor oil | part 2. |

He says it does not set firm like ordinary collodion, but it sets sufficiently to hold its place and to protect the skin from the air, which is the great point, and at same time without any injurious or uncomfortable pressure upon the part.—*Ex.*

**For Constipation.**—EDITOR MEDICAL WORLD:—In reply to Dr. C. C. Gentry, page 82, February World, I would suggest the following, which has proved valuable to me in habitual constipation:

| | | |
|---|---|---|
| ℞ | Pulv. soc. aloes | 2½ ozs. |
| | Bicarbonate soda | 6 ozs. |
| | Pulv. ginger | 3 drms. |
| | Sp. lavender comp. | 2 ozs. |
| | Water | 1 gal. |

M. Sig. One teaspoonful three times a day after meals, and increase if necessary.

To make the solution, macerate 14 days. Then filter and use. Age improves it.

Nashville, Kans. F. S. PECK, M. D.

**Treatment of Chronic Ortorrhœa.**—Dr. S. S. Reynolds asks for treatment for chronic otorrhœa. I have a good deal of this in my practice and give him the following as my treatment in such cases. I have never yet failed to arrest the discharge in four or five days.

Thoroughly cleanse the ear with warm water and apply:

| | | |
|---|---|---|
| ℞ | Pulverized alum | gr. xl |
| | Distilled water | f. oz. iv |

M. S. Apply with ear syringe night and morning.

Fill the ear at night, after using the above wash, with finely pulverized borax and report.—*Ex.*

**Psoriasi Capitis.**—Stern recommends for psoriasis capitis: Præcipit. alb., 10.0; sapon. nigr., 40.0; lanoline anhyd., 50.0 *M. Ft. ung. S.* Rub in every evening a portion the size of a nut.

After four days all the scales are gone, and the affected parts become smooth and take on a normal appearence. It is usually advisable to continue the application of the lanoline alone for a time longer. —*Med. Chir. Rundschau*, 1889.

**Catarrh.**—Carbolic acid and alcohol each ten parts, aqua ammonia twelve parts, and listerine twenty parts. A two-ounce wide mouthed bottle is filled one-third with this mixture, and enough absorbent cotton introduced to absorb the whole. The bottle is then corked and is ready for use by inhalation.—*Med. Brief.*

**Laxative Powder.**—Dr. Dujardin Beaumetz recommends the following as a pleasant and reliable laxative:

| | | |
|---|---|---|
| ℞ | Powdered senna pods (treated with alcohol)<br>Sublimate of sulphur | ää ℥ jss |
| | Powdered fennel<br>Powdered star aniseed | ää grs. 48 |
| | Pulverized cream of tartar | grs. 31 |
| | Powdered liquorice | ℥ ij |
| | Powdered sugar | ℥ vjss. |

Dose. One dessertspoonful of the powder in a half wineglassful of water at night.—*L'Abeille Medicale*, April 27, 1889.

**Hectic Fever.**—For the irritative fever of phthisis pulmonalis, when treatment is absolutely necessary, Prof. Da Costa recommends:

| | | |
|---|---|---|
| ℞ | Antipyrin | gr. ij. |
| | Quininæ sulph. | gr. j. |

Ft. j. in capsul.

M. Sig. One every few hours.

